# Editorial for the Special Issue: “Feature Papers in Drug Toxicity”

**DOI:** 10.3390/toxics12020132

**Published:** 2024-02-06

**Authors:** Youssef Sari

**Affiliations:** Department of Pharmacology and Experimental Therapeutics, College of Pharmacy and Pharmaceutical Sciences, The University of Toledo, Toledo, OH 43614, USA; youssef.sari@utoledo.edu

## 1. Introduction

This editorial introduces the Special Issue “Feature Papers in Drug Toxicity”. Drug toxicity is a major problem involving neurotoxicity and organ injury. There are many drugs, which have toxic effects, as a result of acute or chronic exposure as well as prenatal exposure. These drugs include abused and other drugs that have toxic effects in the brain and other organs of the body. Understanding the pharmacological and toxicological nature of these drugs expand research in the field of toxicology. In this Special Issue, several abused drugs were tested in animal models, including ethanol, hydrocodone, and heroin. In addition, the toxicological effects of other drugs are reported in this Special Issue, including pentylenetetrazol, metronidazole, paracetamol, paclitaxel, and tilmicosin. Importantly, several protective agents were tested by researchers and are reported in this Special Issue. These agents protect against the toxic effects induced by abused and other drugs. In addition, studies report the protective effects of certain plant extracts against drug-induced toxicity.

## 2. An Overview of Published Articles

Costa et al.’s article (**contribution 1**) reports that carbetocin, an analog of oxytocin, attenuated ethanol-induced sensitization behavior in mice of both sexes. This effect was not associated with any changes in the plasma corticosterone concentrations. It is suggested that pharmacological manipulation of oxytocin neurotransmission (using carbetocin for example) and genetic overexpression of the oxytocin receptor may reduce alcohol dependence [1]. This latter study suggests that targeting the oxytocin receptor may have beneficial effects in treating drug dependence (e.g. alcohol). Studies are warranted to investigate the pharmacological mechanism of action mediating the oxytocin receptor in modulating alcohol dependence. 

The article by Wong and Sari (**contributor 2**) reports the therapeutic effect of targeting a major astrocytic glutamate transporter type 1 (GLT-1) on attenuating the effects of chronic exposure to hydrocodone. Thus, this study reports that ceftriaxone, a beta-lactam antibiotic known to upregulate GLT-1, attenuated hydrocodone-induced downregulation of GLT-1 and cystine/glutamate antiporter (xCT) expression and altered signaling kinase pathways such as Akt, Erk, and JNK. It is well known that beta-lactams (e.g. ceftriaxone) have been effective in attenuating the effects of abused drugs, including opioids and alcohol, and these are suggested to be mediated through the upregulation of GLT-1 and xCT expression in the target brain reward regions [2]. Regulation of glutamate homeostasis is critical in drug dependence. Our lab has been working for decades in this field of research and has reported several findings showing that upregulating GLT-1 expression with beta-lactams can lead to attenuation of dependence to abused drugs, including ethanol, cocaine, opioids, methamphetamine, and nicotine. 

Elshebiney et al. (**contributor 3**) reports the benefit of natural polyphenols (e.g. resveratrol, quercetin, magnolol, and β-catechin) on attenuating reinstatement to heroin-seeking behaviors as well as certain neuroinflammatory markers such as IL-6 and TNF-α. In accordance, it has been reported that resveratrol reduced ethanol-induced oxidative stress [3]. This suggests that reducing neuroinflammation with polyphenols may be a key factor in helping to reduce dependence on abused drugs, including alcohol.

Alrashdi et al. (**contributor 4**) reports the effects of Rosmarinus officinalis extract in a pentylenetetrazol-induced epilepsy rat model. The extract from this plant seems to have an inhibitory effect by reducing several seizure symptoms induced by pentylenetetrazol. It is important to note that this plant comprises many phytocompounds or bioactive molecules, which may have therapeutic effects, including the inhibitory effect, which may counterbalance the overexcitation observed in a seizure model [4]. 

The article by El-Moslemany et al. (**contributor 5**) reports that extracts from anise seeds, which are rich in polyphenols, reduced the neurotoxic effects caused by metronidazole. In accordance, pretreatment with anise extracts induced neuroprotective effects in rat model of cerebral ischemia [5]. This suggests that consuming anise seeds may protect against the effects of exposure to certain toxic chemicals or medications as well as against potential neurovascular injury. 

Elgendy et al. (**contributor 6**) revealed that Rhodiola Rosea extracts may protect against tilmicosin-induced cardiotoxicity. Tilmicosin is an antibiotic, which was shown to induce cardiotoxicity in an animal model [6]. The Rhodiola Rosea extracts have been suggested to be effective in alleviating many health issues, including cardiac issues [7]. There are constituents in this plant that may have antioxidant, antiapoptotic, anti-inflammatory and other beneficial health effects. 

The article by Hammad et al. (**contributor 7**) reports the efficacy of vitamin E in protecting against acute exposure to paracetamol during pregnancy. It is important to note that vitamin E has been suggested to have many health benefits, including antioxidant effects during pregnancy [8]. Thus, vitamin E might be taken as supplement during pregnancy to prevent any toxics effects that might be caused by certain medications or other chemicals. 

The article by Elfarnawany and Dehghani (**contributor 8**) reports the toxic effects of paclitaxel, a chemotherapeutic agent, on non-neuronal cells of the rat primary dorsal root ganglia. Paclitaxel was reported to induce neurotoxic effects, including peripheral neuropathy [9]. This latter study suggested that the doses of paclitaxel could be reduced to prevent some of these side effects. 

The last research article in this Special Issue, reported by Abdrabou et al. (**contributor 9**), showed that treatment with metronidazole, an antiprotozoal and antibacterial agent, during pregnancy induced teratogenicity and hepatoxicity and other fetal defects. Thus, metronidazole should be avoided during pregnancy, as another study showed the teratogenic effects with this compound in an animal model [10].

In this Special Issue, one review article by Mai et al. (**contributor 10**), focused on the poisoning effects of Xylazine in humans. The forensic perspectives were also discussed. Xylazine is used as a veterinary drug to induce sedation, analgesia, and muscle relaxation, however, it use in humans can be harmful, particularly with the co-use of other abused drugs, including opioids [11]. 

## 3. Conclusions 

In Summary, this Special Issue reports several findings from investigators in different areas of drug toxicity. The effects of several abused drugs (e.g. ethanol, hydrocodone, and heroin) are reported, and the attenuating effects of certain drug targets were also reported. Other research studies report the toxic effects of certain drugs and medications (e.g. pentylenetetrazol, metronidazole, paracetamol, paclitaxel, and tilmicosin) and the therapeutics effects of certain agents and plant extracts in animal models. 
